# Association of preoperative variables of ipsilateral hip abductor muscles with gait function after total hip arthroplasty: a retrospective study

**DOI:** 10.1186/s42836-022-00126-7

**Published:** 2022-07-01

**Authors:** Tadashi Yasuda, Satoshi Ota, Shinnosuke Yamashita, Yoshihiro Tsukamoto, Eijiro Onishi

**Affiliations:** Department of Orthopaedic Surgery, Kobe City Medical Center General Hospital, 2-1-1 Minatojimaminami-machi, Chuo-ku, Kobe, 650-0047 Japan

**Keywords:** Gait function, Gait speed, Hip abductor, Hip joint, Muscle composition, Timed Up-and-Go test, Total hip arthroplasty

## Abstract

**Background:**

This study aimed to identify the association of preoperative variables of ipsilateral hip abductors with gait function after total hip arthroplasty (THA).

**Methods:**

This study enrolled 42 patients who underwent unilateral primary THA for osteoarthritis. Gait speed and Timed Up-and-Go test were conducted 6 months postoperatively. Preoperative composition of the glutei medius and minimus and the upper portion of gluteus maximus was evaluated by computed tomography. Cross-sectional area ratio of individual composition to the total muscle was calculated. Preoperative variables associated with gait speed and Timed Up-and-Go test after THA were identified by using stepwise regression analysis.

**Results:**

Faster gait speed and shorter Timed Up-and-Go test correlated with smaller cross-sectional area of low-density lean tissue or intramuscular adipose tissue (low-density lean tissue plus intramuscular fat) in the glutei medius and minimus and lower cross-sectional area ratio of low-density lean tissue to the total glutei medius and minimus. Faster gait speed and shorter Timed Up-and-Go test also correlated with larger cross-sectional area of lean muscle mass in the gluteus maximus, higher cross-sectional area ratio of lean muscle mass to the total gluteus maximus, and lower cross-sectional area ratio of intramuscular fat or intramuscular adipose tissue to the total gluteus maximus. Faster gait speed additionally correlated with larger total cross-sectional area of the gluteus maximus. Regression analysis showed that the total cross-sectional area of the gluteus maximus and the low-density lean tissue cross-sectional area of the glutei medius and minimus were the explanatory variables of gait speed and Timed Up-and-Go test after THA, respectively.

**Conclusions:**

There was a potential association between preoperative composition of ipsilateral hip abductors and gait function 6 months after THA. This study indicates a predictive role of preoperative assessment of ipsilateral hip abductor composition in the recovery of gait function after THA.

## Background

Total hip arthroplasty (THA) is an effective treatment for patients with osteoarthritis of the hip. Postoperative functional improvement occurs within the first 6 months [1]. However, approximately 10% of patients report dissatisfaction after THA due to insufficient improvement [2]. It remains unclarified what preoperative factors are associated with functional limitations after THA.

Because gait is one of the most basic movements in daily life, it is necessary to evaluate the recovery of gait function as an outcome after THA. Gait speed has recently received attention as a critical factor for predicting prognosis after THA [3]. In addition, Timed Up-and-Go test (TUG) is widely used to evaluate mobility function in elderly individuals. The Osteoarthritis Research Society International guidelines recommend a 10-m fast-paced walk test for gait speed and TUG as the core set of performance measures for patients with osteoarthritis [4].

A recent systematic review has revealed the association of age and body mass index (BMI) with the functional outcome after THA [5]. There is also evidence on the association between hip abductor muscle function and physical function after THA [6]. In patients with advanced hip osteoarthritis, the gluteus maximus, the gluteus medius and the gluteus minimus are significantly smaller in the symptomatic limb than in the asymptomatic one [7]. In addition to muscle size, the structural muscle composition is an important factor underlying muscle strength and physical function [8]. High association is found between muscle strength and lean muscle mass (LMM) [9]. In elderly people, a loss of LMM can cause a loss of muscle strength [10] and the adipose tissue beneath the deep fascia of a muscle, intramuscular adipose tissue (IMAT), may also explain age-associated muscle strength deficit. Alteration in muscle fiber orientation by fatty infiltration into skeletal muscle decreases force-producing capacities of the whole muscle. Therefore, increased IMAT with loss of LMM could contribute to decline in mobility function in older adults [11]. Fatty infiltration in the gluteus medius is observed concomitantly with minor fatty infiltration in the gluteus minimus in patients with end-stage hip osteoarthritis [12]. However, it is still unclear how hip abductor composition affects functional outcomes after THA. The aim of this study was to evaluate preoperative variables of hip abductor muscles of the operated limb, and elucidate their association with gait function after THA.

## Patients and methods

### Patient selection

We retrospectively analyzed the data from 172 patients who underwent primary THA between November 2018 and June 2020 in our hospital. The exclusion criteria were: (1) history of total knee arthroplasty (29 patients), (2) contralateral THA within 6 months before admission (53 patients), (3) a hip surgical procedure on the operated side (3 patients), (4) hip deformity with Crowe types 2, 3, and 4 (14 patients) [13], (5) bilateral THA (1 patient), (6) contralateral pain with hip or knee osteoarthritis (25 patients), (7) any other musculoskeletal impairments affecting lower limb function (1 patient), and (8) insufficient clinical data (4 patients). As a result, 42 of 172 patients were enrolled into this study. Of the 42 patients, 8 patients had undergone contralateral THA over a period of 12 months before admission. All patients were able to walk independently, with or without a cane, before surgery. Mean age and BMI of the 42 patients (9 males and 33 females) were 70.9 years (range, 46–87 years) and 22.8 kg/m^2^ (range, 15.2–29.8 kg/m^2^), respectively.

### Operation and postoperative rehabilitation

THA was performed through a lateral approach with modified Mostardi technique for minimal damage to the hip abductor muscles [14]. After blunt dissection through the anterior one-fourth of the gluteus medius, the bony portion that retains the tendinous junction of the glutei medius and minimus was osteotomized using a chisel. The osteotomized trochanteric fragment, which was approximately 10 mm long, 10 mm wide and 5 mm deep, was mobilized anteriorly and medially, whereas the vastus lateralis was kept completely intact. Finally, the osteotomized trochanteric fragment was anatomically re-attached by inducing bone-to-bone contact using ultra-high molecular-weight polyethylene sutures. The same rehabilitation protocol was used in each inpatient during the first 2 weeks in our hospital and thereafter 3–4 weeks in the recovery-phase rehabilitation hospital following THA. A standard rehabilitation program started on the first postoperative day, and patients were allowed to eliminate walking aids whenever comfortable. Physical therapy included therapies designed to progressively improve walking ability and other functional activities, and stairs-walking according to the needs and progress of each patient. Patients participated in a progressive program involving range of motion exercises, strengthening exercises, and functional training. No patient received outpatient physical therapy.

### Muscle composition

**Fig. 1:**
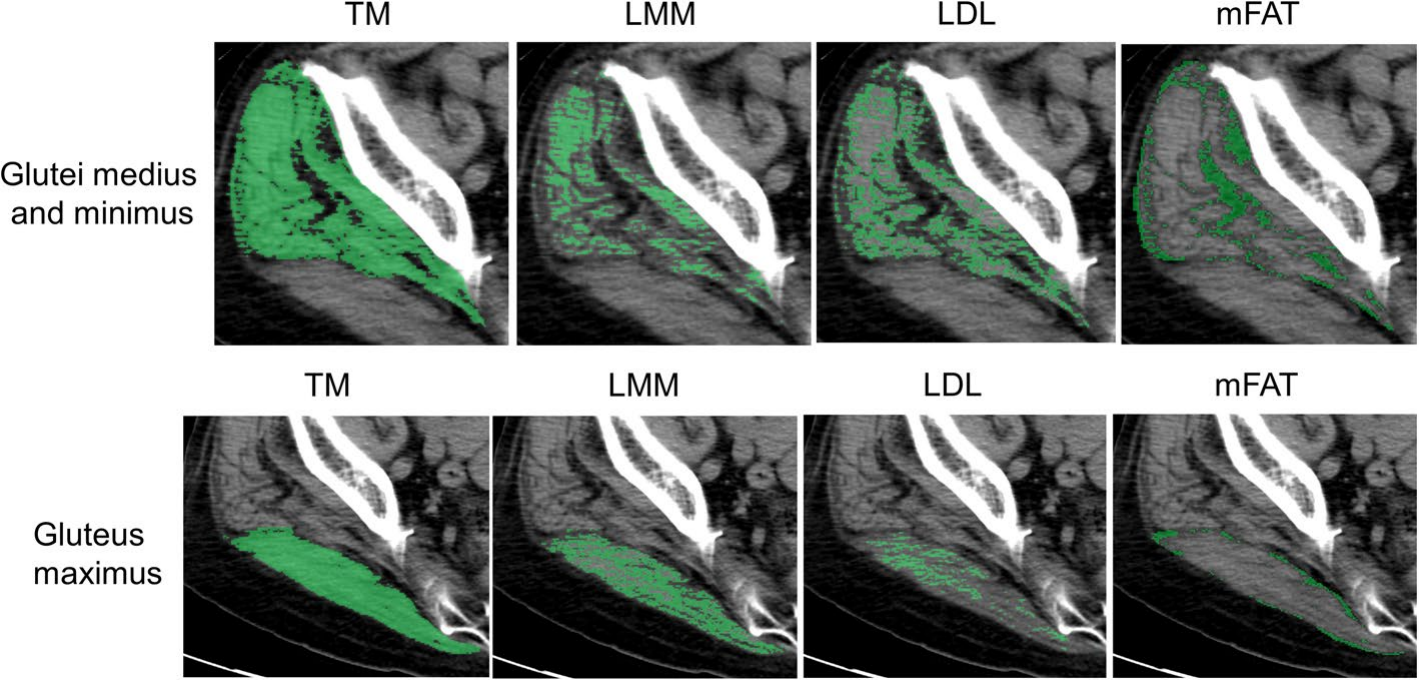
Measurement of muscle composition on an axial image of computed tomography. Total muscle cross-sectional area (TM) of the glutei medius and minimus or the upper portion of the gluteus maximus is segmented using the threshold of − 29 to 150 Hounsfield units (HU). Lean muscle mass (LMM), low-density lean tissue (LDL), and intramuscular fat (mFAT) are colored as the pixels with the density of 30 to 80 HU, 0 to 29 HU, and − 190 to − 30 HU, respectively, within each segmented TM

Muscle composition of the operated limb was evaluated by computed tomography (CT) performed for preoperative planning within 2 weeks before THA. Muscle composition of the glutei medius and minimus and the gluteus maximus was analyzed on a single axial CT slice at the bottom end of the sacroiliac joint [15]. The upper portion of the gluteus maximus originates from the posterior iliac crest, while the lower portion of the gluteus maximus arises from the inferior sacrum and upper lateral coccyx [16]. Thus, the upper portion of the gluteus maximus was evaluated almost exclusively at this level. Those muscle groups were manually outlined and thereafter automatically segmented based on attenuation values: − 29 to 150 Hounsfield units (HU) using SYNAPSE VINCENT software (version 5.0, Fujifilm Co., Tokyo, Japan), according to our previous study [17]. The software electronically calculated the cross-sectional area, in cm^2^, of the segmented total muscle group (TM). High-density lean tissue that comprises little fatty infiltration is recognized as a measure of LMM. Elevated levels of adipocytes between and within muscle fibers in low-density lean tissue (LDL) result in decreased CT density compared with LMM. Intramuscular fat (mFAT) shows the lowest CT density. Cross-sectional areas of LMM, in cm^2^, LDL, and mFAT within each TM were measured electronically with the software as the areas of pixels according to the definition by attenuation values: 30 to 80 HU for LMM, 0 to 29 HU for LDL, and − 190 to − 30 HU for mFAT [17, 18]. Representative images are shown in Fig. 1. IMAT was defined as the summation of the areas of both LDL and mFAT [17, 18]. As shown in our previous study [17], the intra-class correlation coefficient (ICC) was 0.98–0.99 for muscle composition measurement. The area of TM or each component was normalized for the square of the patient’s height (cm^2^/m^2^). Alternatively, LMM, LDL, mFAT, and IMAT were normalized for the size of respective muscle by calculating a percentage of each measure relative to TM [17, 18], designated as LMM/TM, LDL/TM, mFAT/TM, and IMAT/TM. Because alterations in the place of muscle section by hip deformity may affect cross-sectional CT analysis, this study excluded patients with hip deformity of Crowe types 2, 3, and 4.

### Functional outcome measures

The following measures were assessed at admission and at 6 months postoperatively when functional performance reached a plateau after THA [1]. Gait speed was evaluated by timing a patient instructed to walk as quickly and safely as possible across a 10-m course with 2-m acceleration and deceleration zones [4, 17]. Gait speed (cm/s) was measured twice, and the faster speed was used for analysis. TUG required a patient to rise from an armed chair, walk 3 m, turn around, and return to sit on the chair. This test was timed in seconds and the faster of the 2 trials was used for analysis [17, 19]. Isometric muscle strength of ipsilateral hip abductor was measured using a hand-held dynamometer as described previously [20]. ICC ranged from 0.93–0.96 for measurement of maximal isometric strength.

### Statistical analysis

The normality of data was assessed by Shapiro-Wilk test. Pairwise comparisons were made between preoperative and postoperative gait function or muscle strength by a 2-sample Wilcoxon’s rank sum test. Cohen’s d was calculated for the comparison between two means. Pearson’s correlation coefficients were calculated to determine associations between preoperative variables and gait function before and after THA. Stepwise multiple regression analysis was performed to identify significant explanatory variables for gait function after THA. Differences were considered to be statistically significant at *P* <  0.05. All analyses were performed using SPSS (version 25.0, IBM-SPSS, Chicago, IL, USA). Our preliminary estimation of 10 patients with unilateral primary THA found a potential association between preoperative CT variables of ipsilateral hip abductors and gait function, with Pearson’s correlation r = 0.43. Using this preliminary correlation, a priori power analysis by the two-sided bivariate normal model of correlation with G*power software demonstrated that 40 patients would be needed to detect significant differences, assuming a two-tailed type I error rate of 0.05 and a power of 80%. Thus, 42 patients in this study were considered to be sufficient.

## Results

Table 1 shows preoperative muscle composition of the glutei medius and minimus and the gluteus maximus.

**Table 1:** Preoperative muscle composition

Muscle composition	TM (cm^2^/m^2^)	LMM (cm^2^/m^2^)	LMM/TM (%)	LDL (cm^2^/m^2^)	LDL/TM (%)	mFAT (cm^2^/m^2^)	mFAT/TM (%)	IMAT (cm^2^/m^2^)	IMAT/TM (%)
Glutei medius+minimus	13.4 (2.3)	7.5 (2.6)	55.3 (13.1)	3.5 (0.9)	26.7 (7.0)	2.2 (1.3)	17.6 (11.7)	5.7 (2.1)	44.3 (18.0)
Gluteus maximus	9.2 (2.3)	4.4 (2.7)	45.4 (19.8)	3.3 (1.1)	37.1 (11.4)	1.2 (0.7)	14.3 (8.4)	4.6 (1.5)	51.4 (17.8)

Data are expressed as mean (standard deviation). *TM* Segmented total muscle cross-sectional area, *LMM* Lean muscle mass area, *LDL* Low-density lean tissue area, *mFAT* Intramuscular fat area, *IMAT* Intramuscular adipose tissue area

Table 2 demonstrates preoperative and postoperative gait function and hip abductor strength of the operated limb. Gait speed and TUG improved at 6 months after THA compared with the preoperative data. Similarly, ipsilateral hip abductor strength increased postoperatively.

**Table 2:** Postoperative alterations in gait function and muscle strength

	Preoperative	6 months	*P* values	Cohen’s d
Gait speed (cm/sec)				
mean (standard deviation), 95% confidence interval	80.6 (32.6), 70.4, 90.7	105.2 (24.1), 97.7, 112.7	< 0.001	0.85
Timed Up-and-Go test (sec)				
mean (standard deviation), 95% confidence interval	16.7 (16.3), 11.6, 21.8	9.0 (3.5), 7.9, 10.1	< 0.001	0.63
Ipsilateral hip abductor strength (Nm/kg)				
mean (standard deviation), 95% confidence interval	0.41 (0.21), 0.35, 0.48	0.69 (0.23), 0.61, 0.76	< 0.001	1.24

Pearson’s correlations were analyzed between preoperative muscle composition and gait function after operation (Table 3). LDL, LDL/TM, and IMAT of the glutei medius and minimus correlated negatively with postoperative gait speed and positively with postoperative TUG. TM, LMM and LMM/TM of the gluteus maximus correlated positively with gait speed after THA whereas mFAT/TM and IMAT/TM of the gluteus maximus correlated negatively with gait speed after THA. Postoperative TUG correlated negatively with LMM and LMM/TM of the gluteus maximus and positively with mFAT/TM and IMAT/TM of the gluteus maximus.

**Table 3:** Pearson’s correlation coefficients between muscle composition and postoperative gait function

Glutei medius+minimus	TM	LMM	LMM/TM	LDL	LDL/TM	mFAT	mFAT/TM	IMAT	IMAT/TM
Gait speed	−0.02	0.17	0.25	−0.39**	− 0.34*	− 0.25	− 0.22	− 0.33*	−0.28
TUG	0.20	−0.09	−0.24	0.53**	0.33*	0.20	0.13	0.36*	0.21
Gluteus maximus	TM	LMM	LMM/TM	LDL	LDL/TM	mFAT	mFAT/TM	IMAT	IMAT/TM
Gait speed	0.47**	0.44**	0.32*	0.06	−0.25	−0.19	−0.35*	−0.04	− 0.32*
TUG	−0.30	− 0.39*	−0.39**	0.08	0.29	0.22	0.34*	0.15	0.35*

*TM* Segmented total muscle cross-sectional area, *LMM* Lean muscle mass area, *LDL* Low-density lean tissue area, *mFAT* Intramuscular fat area, *IMAT* Intramuscular adipose tissue area, *TUG* Timed Up-and-Go test. Values highlighted in bold are statistically significant (*P <  0.05 and **P < 0.01)

Pearson’s correlations were also calculated between preoperative clinical data and gait function after THA (Table 4). Postoperative gait speed correlated negatively with age. Postoperative TUG correlated positively with age. Preoperative gait speed correlated positively with postoperative gait speed and negatively with postoperative TUG. In contrast, preoperative TUG demonstrated no correlation with postoperative gait function.

**Table 4:** Pearson’s correlation coefficients between preoperative data and postoperative gait function

	Age	BMI	Ipsilateral hip abductor	Preoperative gait speed	Preoperative TUG
Postoperative gait speed	−0.43**	− 0.04	0.22	0.45**	−0.24
Postoperative TUG	0.38*	0.14	−0.18	−0.41**	0.14

*BMI* Body mass index, *TUG* Timed Up-and-Go test. Values highlighted in bold are statistically significant (*P < 0.05 and **P < 0.01)

Table 5 shows the results of stepwise regression analysis using the variables based on the results from Tables 3 and 4. From regression analysis using the 10 significant variables for gait speed at 6 months (Tables 3 and 4), TM of the gluteus maximus was selected as the variable that explained 20.3% of gait speed after THA. Regression analysis using the 9 significant variables for postoperative TUG (Tables 3 and 4) showed that LDL of the glutei medius and minimus was identified as the variable that explained 26.0% of TUG at 6 months.

**Table 5:** Stepwise regression analysis for postoperative gait function

	Explanatory variables	B	SE(B)	β	t-ratio	*P* value	95% CI	Adjusted R^2^
Gait speed	Gluteus maximus TM	0.050	0.015	0.472	3.383	0.002	0.020, 0.080	0.203
TUG	Glutei medius+minimus LDL	2.041	0.521	0.527	3.921	< 0.001	0.989, 3.094	0.260

*B* partial regression coefficient, *SE* Standard error, *β* Standardized partial regression coefficient, *CI* Confidence interval, *R*^*2*^ Coefficient of determination, *TUG* Timed Up-and-Go test, *TM*, segmented total muscle cross-sectional area, *LDL* Low-density lean tissue area

## Discussion

Currently, it remains uncertain what preoperative factors link to suboptimal outcomes after THA. This study evaluated preoperative muscle composition, LMM, LDL, mFAT, and IMAT as combination of LDL and mFAT within TM of the glutei medius and minimus and the gluteus maximus by CT software. Quantification of preoperative muscle degeneration in relation to postoperative gait function has not received sufficient attention. This study provides the first evidence that preoperative hip abductor muscle composition of the operated limb may at least partially correlate with gait function at 6 months after THA when postoperative functional recovery arrives at a plateau [1].

TM of the upper gluteus maximus was found to be the explanatory variable of gait speed with positive correlation after THA. Restriction of hip extension during the late stance phase of gait in patients with osteoarthritis may cause disuse atrophy of the lower gluteus maximus that acts as the primary hip extensor [16]. In contrast, no significant alterations are observed in the upper gluteus maximus that works as the hip abductor [16]. The hip abductors can be divided into superficial muscles like the gluteus maximus with their insertion into the iliotibial band and deeper muscles such as the glutei medius and minimus with their insertion into the greater trochanter. Ipsilateral glutei medius and minimus are smaller in patients with unilateral hip osteoarthritis than in healthy controls [21] or than the contralateral hip abductor [5]. Taken together, the upper gluteus maximus with no significant atrophy could play a critical role in gait speed after THA.

LDL of the glutei medius and minimus could be positively associated with TUG after THA. Recently, fatty infiltration within the skeletal muscles has emerged as a critical factor of muscle quality. IMAT resulting from fatty infiltration may be a predictor of muscle function in older individuals [22]. IMAT in the hip abductors correlates with poor balance and mobility dysfunction compared with IMAT in the distal thigh muscles in older adults [18]. Fatty infiltration into the gluteus medius in end-stage hip osteoarthritis is constantly observed in contrast to its absence in healthy controls [12]. In addition, minor fatty infiltration within the gluteus minimus is also found as a result of normal ageing [12]. Therefore, increased LDL in the glutei medius and minimus, as a part of IMAT, likely has a negative influence on postoperative TUG.

There is a substantial loss of muscular strength in the ipsilateral limb before THA compared with the contralateral limb before operation [23]. However, no association is found between TUG at 7 months after THA and preoperative strength of hip abductor of the ipsilateral limb [20]. There is possibility that preoperative muscle strength of the affected limb fails to reflect the potential function because of pain during muscle strength measurement [24]. In comparison of muscle strength, preoperative muscle quality, like muscle composition of the affected limb, may be highly associated with gait function after THA. CT provides high-quality image reconstruction and stable attenuation values that aid in image segmentation to evaluate muscle composition. Since CT is commonly employed for preoperative planning, preoperative assessment of hip muscle composition by CT could be helpful for prediction of functional recovery after THA. This study indicates a possible association of larger TM of the gluteus maximus and smaller LDL in the glutei medius and minimus with better recovery of gait speed and TUG after THA, respectively. Because low-density muscle could decrease with aerobic exercise training [25], effects of preoperative rehabilitation intervention on muscle composition should be the subject of future investigation.

In addition to CT, magnetic resonance imaging (MRI) is an excellent tool to depict detailed muscle structures. A recent study has shown atrophy and fatty infiltration in the gluteus medius and gluteus minimus in end-stage hip osteoarthritis by MRI [12]. In contrast to quantitative measurement of muscle cross-sectional areas, a semiquantitative grading system originally described by Goutallier *et al*. [26] is commonly used to evaluate fatty infiltration into muscles by MRI. Thus, quantitative analysis by CT density could be suitable for assessment of muscle composition compared with categorical grading evaluation by MRI.

This study has several limitations. First, this was a monocentric retrospective study. All patients received the same surgical technique and postoperative management, which could have influenced the results. Second, there were no data on the muscle composition of the contralateral limb or the lower gluteus maximus. Since preoperative hip abductor composition selected by the regression analyses could explain up to 20–26% of postoperative gait function, there may be other explanatory variables of gait function after THA. Third, the pelvic alignment, the implant position, and other confounding factors were not assessed in this study. Whereas femoral offsets could influence hip abductor strength after THA [27], a recent study has shown that alterations in acetabular and femoral offsets after THA have no effect on postoperative gait function [28]. Fourth, it remains uncertain whether patients with bilateral symptomatic osteoarthritis or severe hip deformity may show similar results. Fifth, muscle composition was evaluated on a single axial CT slice. Although the cross-sectional area of the gluteus medius measured at the inferior point of the sacroiliac joint correlates with both the muscle volume and peak isometric strength [16], measurements in axial CT images are potentially variable and may depend on the place of section. In addition, this study failed to employ adjustment with bone mineral reference phantom, which can ensure greater accuracy in assessing muscle degeneration using CT data [29]. Lastly, skeletal muscle mass is smaller in women than in men [30]. Future studies may need to analyze muscle composition of male and female patients separately.

## Conclusions

This study has provided the first evidence that the cross-sectional area of the upper portion of the gluteus maximus and the area of low-density lean muscle in the glutei medius and minimus of the affected limb before THA are partially associated with gait function after THA. Preoperative predictors potentially assist identification of patients at risk for functional difficulties after THA. Addition of muscle composition to preoperative risk assessment could offer further information to develop a screening instrument for identification of high-risk patients.

## Data Availability

Not applicable.

## Abbreviations

THA: Total hip arthroplasty
TUG: Timed Up-and-Go test
BMI: Body mass index
LMM: Lean muscle mass
IMAT: Intramuscular adipose tissue
CT: Computed tomography
HU: Hounsfield units
TM: Segmented total muscle
LDL: Low-density lean tissue
mFAT: Intramuscular fat
ICC: Intra-class correlation coefficient
MRI: Magnetic resonance imaging
